# Colorectal Stenting in Malignant Large Bowel Obstruction: The Learning Curve

**DOI:** 10.1155/2011/917848

**Published:** 2010-10-11

**Authors:** D. Williams, R. Law, A. M. Pullyblank

**Affiliations:** ^1^Department of Surgery, North Bristol NHS Trust, Frenchay Hospital, Frenchay Park Road, Bristol, BS16 1LE, UK; ^2^Department of Radiology, North Bristol NHS Trust, Frenchay Hospital, Frenchay Park Road, Bristol, BS16 1LE, UK

## Abstract

*Aim*. Self-expanding metal stents (SEMSs) are increasingly used for the palliation of metastatic colorectal cancer and as a bridge to surgery for obstructing tumours. This case series analyses the learning curve and changes in practice of colorectal stenting over a three year period. *Methods*. A study of 40 patients who underwent placement of SEMS for the management of colorectal cancer. Patients spanned the learning curve of a single surgeon endoscopist. *Results*. Technical success rates increased from 82% initially, using an average of 1.7 stents per procedure, to a 94% success rate where all patients were stented using a single stent. There has been a change in practice from elective palliative stenting toward emergency preoperative stenting. *Conclusion*. There is a steep learning curve for the use of SEMS in the management of malignant colorectal bowel obstruction. We suggest that at least 20 cases are required for an operator to be considered experienced.

## 1. Introduction

Up to 29% of the 40 000 new diagnoses of colorectal cancer each year in the UK present with obstructive symptoms [1, 2]. Self-expanding metal stents (SEMSs) are being used with increasing success for the palliation of metastatic colorectal cancer and as a bridge to surgery for obstructing tumours despite the absence of high level evidence for their safety and efficacy. With the advent of the ColoRectal Stenting Trial (CReST trial), a multicentred RCT, there has been debate as to the learning curve for colorectal stenting. This case series investigated the first three years of a single surgeon endoscopist stenting experience analysing the change in practice over time and the learning curve.

## 2. Patients and Methods

Data was collected prospectively on 40 patients presenting with acute or subacute large bowel obstruction that subsequently underwent the insertion of an SEMS. There were 2 indications for insertions of an SEMS in this case series. Firstly, patients with disseminated malignancy from a lesion in the left colon who were experiencing obstructing symptoms were considered. The decision to attempt insertion of SMES was taken in the MDT. These cases with subacute large bowel obstruction were stented for palliation only. The second indication for stenting was acute left-sided large bowel obstruction. Stenting was indicated in this cohort if the patient was at high risk for emergency surgery or had disseminated disease. Consecutive eligible patients were selected over a three-year period (2006–2008). 

**Figure 1:**
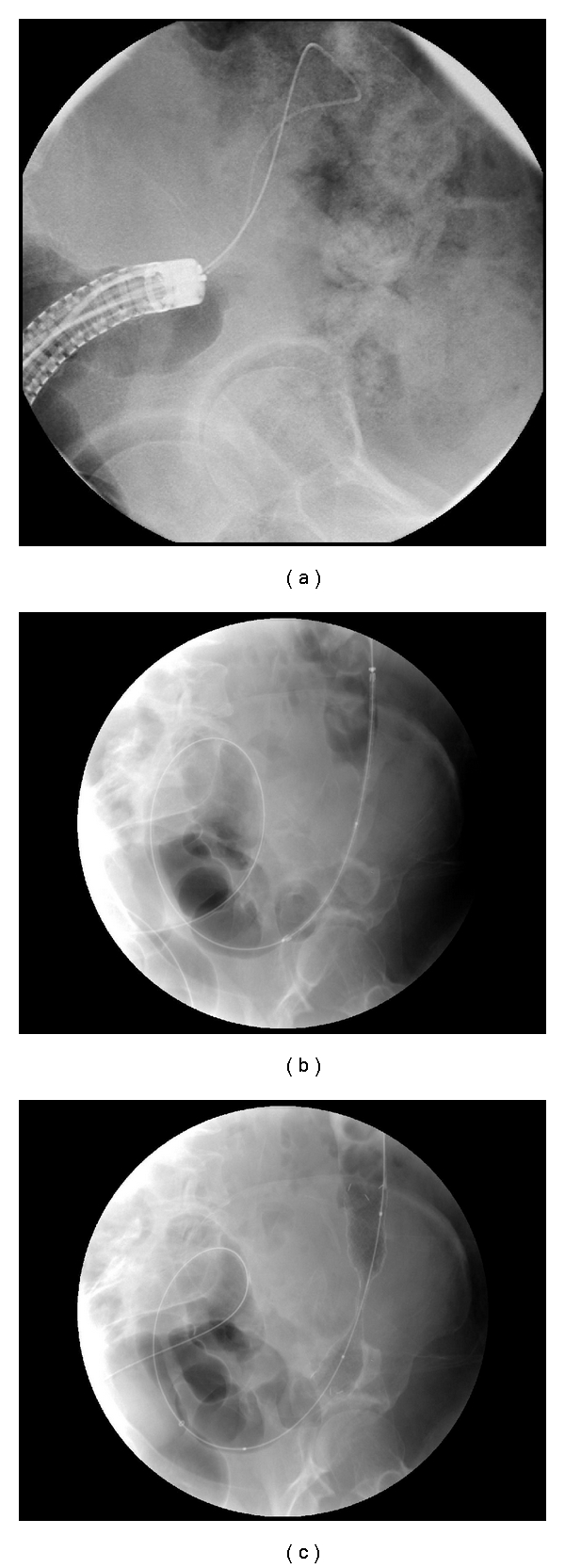
A series of images demonstrating the stages involved in endoscopically guided colorectal stenting. (a) The tumour is directly visualised with the endoscope, and the guide wire is passed through the end of the scope. (b) The deployment system is passed through the stricture. (c) The stent has been successfully deployed. The stent boundaries can be clearly identified.

The procedures were performed by a single surgeon endoscopist and a consultant radiographer in one centre. Stents were performed under combined endoscopic and radiological guidance (Figure 1). Technical success was defined as successful placement of the SEMS across the obstructing lesion with good passage of contrast post stenting. In this series, technical success equated to clinical success and all patients who achieved a technical success had symptomatic relief. 

Three patients were excluded at the time of the procedure as stenting was thought inappropriate (stenting was not attempted). One patient had a pre-existing perforation demonstrated with contrast and preceded with operative intervention. Endoscopy and radiological studies failed to detect an obstructing malignant lesion in the other two patients, and stenting was not performed. 

This paper analysed the resulting 37-patient case series. To aid with analysis and to show a change in practice over time, the series was divided chronologically in half (1st 18 patient were stented in 2006 and 2nd 19 patients stented in 2007 and 2008). The site of the lesions stented were 6 descending colon, 9 sigmoid colon, 12 rectosigmoid, 8 rectum, 1 extrinsic compression, and 1 at the splenic flexure.

## 3. Results

Thirty seven patients underwent stent placement (22 male, 15 Female) with an average age of 74 (55–95). 18 (49%) patients presented as an emergency with acute large bowel obstruction. Of these patients, the staging investigation demonstrated disseminated disease in 11 cases. After discussion in the MDT all these patient were subsequently managed palliatively (nonoperatively). The remaining 7 had localised disease and were stented as a bridge to surgery. The other 19 patients presented with subacute bowel obstruction and were electively stented. All of these patients were later managed with palliation for disseminated disease. Over 80% of the patients in this series were managed with palliation. As a result of this they did not undergo surgical resection and formal histological (TNM) staging.

The site of lesion and stenting varied: 6 descending colon, 9 sigmoid colon, 12 rectosigmoid, 8 rectum, 1 extrinsic compression, and 1 at the splenic flexure. Complications included 4 failures (11%), 4 tumour overgrowths (11%), and 1 stent migration (3%). Three of the tumour overgrowth cases were restented at day 22, 130, and 146 after the original procedure. One patient had a late tumour overgrowth which was managed with a defunctioning loop ileostomy. There was 1 early perforation and 1 late (2 weeks) perforation in 2 of the patients that were restented. Both these patients died as they were not fit for surgery. 

**Figure 2:**
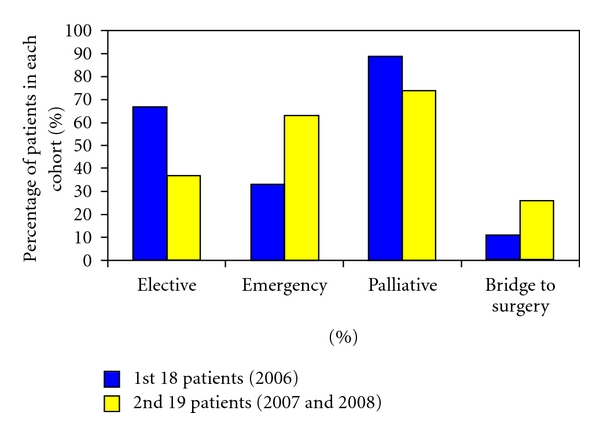
A comparison between the first and second half of the case series showing the change in practice over time.

Figure 2 demonstrates the difference between the patients in the first and second half of the case series. The early patients were predominantly palliative patients electively admitted with subacute large bowel obstruction. 67% in the first 18 patients and 37% in the subsequent 19 patients were electively stented for this indication. In the second half of the series, more SEMS are deployed acutely as a bridge to surgery or to palliate emergency large bowel obstruction. Of the first 18 patients only 11% of patients were stented following emergency presentation with acute large bowel obstruction, in comparison to 26% in the subsequent 19 patients.

The first 11 stents performed had an 82% technical success rate and required an average of 1.7 stents per procedure. The subsequent 10 stents performed had a 90% technical success rate and required average of 1.1 stents per procedure. The final 16 stents performed had a 94% technical success rate. All of these patients were stented using a single stent. This is shown in Table 1 demonstrating how technical success increased and number of stents decreased with experience.

## 4. Discussion

Colorectal cancer commonly presents with large bowel obstruction, often at an advanced stage with only 50% of patients being suitable candidates for curative surgery. Emergency surgery in these patients has a high mortality and morbidity when compared to elective surgery. Over time, several techniques such as balloon dilatation and laser ablation have been attempted with the aim of decompressing the bowel. These techniques had limited success. The use of stents in colorectal obstruction was first reported in the early 1990s [3, 4] and subsequently has been used to avoid surgery in patients with metastatic disease and as a bridge to surgery in those with localised disease.

SEMS have many advantages for patients with acute bowel obstruction. An SEMS can be used to control an emergency presentation allowing patient optimisation. A study of 8000 patients found that emergency surgery has a mortality of 19.3% compared to 5.6% for elective surgery [5]. Elective surgery also reduces morbidity, with higher rates of primary anastomosis and lower rates of severe complications. Martinez-Santos et al. found that patients undergoing emergency surgery for acute malignant large bowel obstruction had a primary anastomosis rate of 41% compared with 87% in patients operated on following insertion of an SEMS [6], thus saving a significant amount of stoma-related morbidity. In the long term, only 60% of patients with a colostomy proceed to stoma reversal [7]. In patients having curative surgery following a bridge to surgery SEMS, up to 95% go onto having a single staged procedure avoiding a colostomy [8].

There are also health economic advantages [8] with reduced hospital stay [9, 10] and length of time in critical care beds [6, 11]. Importantly, stenting can buy time for staging, treatment planning, neoadjuvant therapies and patient optimisation. In this case series 18 patients presented with acute bowel obstruction and underwent emergency stenting. Of these patients, 11 (61%) were found to have disseminated disease at staging and hence avoided unnecessary major surgery.

Colorectal stenting itself has disadvantages. The majority of the published literature is limited by having a small sample size and being nonrandomised; consequently there is a large range in quoted complication rates. A review by Watt et al. found median complication rates of stent migration 11%, perforation 4.5%, and tumour overgrowth 12% [12]. Clinical success is usually quoted at a rate of 85–100% [1, 8, 12, 13] with mortality between 0 and 2%. This study had a primary perforation rate of 0% in line with the literature, but we had an overall mortality of 2/37 (5%) when restented patients were taken into account. Successful treatment of acute left-sided colorectal obstruction depends on a number of operator and patient factors. A Cochrane review in 2002 concluded that the limited number of randomised control trials into the management of obstructing left-sided colorectal carcinoma together with methodological weaknesses does not allow reliable assessment of the best treatment strategy [14]. There is a clear need for further large randomised studies.

The only prospective multicentred RCT to date, the Stent-in 1 study [15, 16], was stopped early following a high rate of stent-related complications. In the proposed followup Stent-in 2 study [17], it is suggested that SEMS should be placed by an experienced gastroenterologist. They defined an experienced gastroenterologist as one who has placed 20 enteral stents of which at least 10 were colonic, without giving evidence why these figures have been suggested. This study found that there was a definite learning curve for the insertion of SEMS, the first 11 stents having only an 82% success rate, the next 10 having a 90% success rate, and the subsequent SEMS having a 94% success rate. In this case series, it was also noted that the number of SEMS required to relieve an obstruction decreased with experience. Initially an average of 1.7 stents were used per successful procedure whereas towards the end of the series strictures were consistently requiring only one stent. This increased stent usage early in the series reflected inexperience and technical difficulty rather than length of stricture. There was no significant difference between complications and length of stricture between the groups. This evidence suggests that operators should have a minimal experience of 20 colonic SEMS to be eligible for inclusion in future RCTs. 

Not being a randomised study, there is inevitable bias in the selection of patient and treatment options. Experience in colorectal stenting resulted in changes in practice over time. As the study progressed, more stents were performed on an emergency basis (33% of the first 18 patients and 63% of the second 19 patients) in a patient demographic who were likely to be more unwell. More stents were also performed as a bridge to surgery in the second half of the study (26% compared with 11%). This change in practice is likely to reflect an increase in operator confidence in their technical ability and knowledge of when stenting is and is not appropriate. The fact that the clinical success rates increased along this learning curve despite more technically difficult patients suggests that there may also be a significant learning curve in patient selection. 

There has also been a change in practice with regard to restenting. The 2 mortalities in this series were patients who died following the restenting of a multilevel obstruction. Both these patients required 2-3 stents to relieve the obstruction caused by a long stricture, which in retrospect was a mistake. If a reobstruction cannot be solved with a single stent, practice has changed and a further stent would not be attempted. 

In conclusion this case series suggests that initially there is a steep learning curve for the use of SEMS in the management of malignant colorectal bowel obstruction of about 11 cases. We suggest that at least twenty cases are required for an operator to be considered experienced.

## Figures and Tables

**Table 1:** The learning curve for SEMS.

Stenting group	Percentage success (%)	*N*° of stents per procedure
1–11	82	1.7
12–21	90	1.1
22–37	94	1.0
